# A Novel Bilinear Feature and Multi-Layer Fused Convolutional Neural Network for Tactile Shape Recognition

**DOI:** 10.3390/s20205822

**Published:** 2020-10-15

**Authors:** Jie Chu, Jueping Cai, He Song, Yuxin Zhang, Linyu Wei

**Affiliations:** School of Microelectronics, Xidian University, Xi’an 710071, China; jiechu@stu.xidian.edu.cn (J.C.); songhe@stu.xidian.edu.cn (H.S.); yxzhang_77@stu.xidian.edu.cn (Y.Z.); lywei@stu.xidian.edu.cn (L.W.)

**Keywords:** tactile shape, convolutional neural network, bilinear feature, multi-layer fusion

## Abstract

Convolutional neural networks (CNNs) can automatically learn features from pressure information, and some studies have applied CNNs for tactile shape recognition. However, the limited density of the sensor and its flexibility requirement lead the obtained tactile images to have a low-resolution and blurred. To address this issue, we propose a bilinear feature and multi-layer fused convolutional neural network (BMF-CNN). The bilinear calculation of the feature improves the feature extraction capability of the network. Meanwhile, the multi-layer fusion strategy exploits the complementarity of different layers to enhance the feature utilization efficiency. To validate the proposed method, a 26 class letter-shape tactile image dataset with complex edges was constructed. The BMF-CNN model achieved a 98.64% average accuracy of tactile shape. The results show that BMF-CNN can deal with tactile shapes more effectively than traditional CNN and artificial feature methods.

## 1. Introduction

Tactile perception is relayed to bionic machines through an electronic skin by transforming external stimuli into sensitive component output changes, thus improving human–computer interaction intelligence [1]. The tactile shape is one of the major physical properties of objects and has been widely used as an important recognizable target in numerous and different fields, including human–computer interaction, intelligent medical treatment, and wearable devices [2,3]. 

In shape recognition tasks, the pressure information collected by the sensor array is treated as an image for subsequent processing [4,5,6]. Thus, many artificial design approaches that achieve outperformance in the visual domain have been transferred to tactile shape perception. Lou et al. established a dataset consisting of 18 real objects including combs, balls, cups, etc., and proposed a tactile-SIFT method to extract features, which achieved 91.33% accuracy [7]. Gandarias et al. developed a tactile-SUFR descriptor and trained a support vector machine (SVM) classifier to recognize 5-class objects and 3-class human body parts, and achieved 80% accuracy and fast processing time [8]. Khasnboish et al. applied chain codes to describe the tactile shape of eight woody geometries and four irregular shaped household objects, and a compositive SVM was employed to classify with an average classification accuracy of 93.46% [9]. Through a literature review, it can be found that these artificial local feature-based methods are capable of accomplishing tactile recognition. However, such feature design processes are labor-intensive because the description of features requires researchers to analyze the raw information and consider various invariants, and then manually design them for a specific application. To improve this situation, end-to-end semantic feature approaches have been proposed that automatically extract features and complete the classification [10]. One of the most prominent methods is the use of convolutional neural networks (CNNs). Due to their excellent 2D and 3D information extraction capabilities, CNNs are suitable for extracting tactile features [11,12,13,14]. Gandarias et al. applied several CNN models, including eight transfer learning-based models and three custom-made models to identify 22-class contacted household objects using a high-density sensor array, and achieved over 95% accuracy [15]. Pastor et al. acquired pressure images at different grip forces during active perception in order to form a 3D tactile tensor, which was then fed to 3DCNN. A recognition rate of 98% was obtained on a self-constructed dataset consisting of 24 categories of objects [16]. Cao et al. proposed a residual orthogonal tiling and pyramid convolution ensemble method for tactile recognition based on a robotic arm, and achieved 97.5% accuracy on the HCs-10 dataset [17]. Tsuji et al. constructed a pen-type tactile system, inputting time and amplitude sequences as 2D images, and obtained a roughness recognition accuracy of 82.1% for a single user [18]. Hui et al. developed a fusion model of CNN and long short term memory (LSTM) network. A self-built 14-category of common objects was tested for 14 classifications and four classifications, whose accuracy rates were 94.2% and 95%, respectively [19]. Funabashi et al. used morphology-specific convolutional neural networks for tactile object recognition with a multi-fingered hand and seven different combinations of variations were evaluated; an object recognition rate of over 95% with 20 objects was achieved [20]. Alameh et al. transformed 3D tensorial tactile data into 400 × 400 RGB images, and used multiple mainstream deep CNNs for 3-class touch modalities recognition through transfer learning methods, in which the inception-resnet method achieved the highest recognition rate of 76.9% [21].

However, tactile shape perception remains challenging, which can be attributed to the fact that the raw signal from the sensor inevitably suffers from the following non-ideal effects. Firstly, the density of sensors cannot be comparable to vision, at only ~10^2^/cm^2^ [22]. The resulting tactile images have a low resolution. Thus, the raw shape mapping is inadequate. Additionally, current mainstream neural networks are based on visual images and enhanced learning ability by deepening and widening the structure, and CNNs are no exception [23,24,25]. However, the tiny size and low resolution of tactile images limit the depth of configurable CNNs. Secondly, due to the requirement of flexibility, sensors are made of a low modulus elastic material [26]. Because of the unavoidable elastic coupling, the unpressed pixels around the pressed pixels are prone to deform during the touch action, generating pseudo outputs [27]. This leads to edge blurring, which is especially intensified in shapes with complex contours. Thus, the raw information is mapped inaccuracy. As a result, low resolution and edge-blurred tactile images are learned inadequately, leading to their misrecognition.

We explore how to improve the performance of tactile shape recognition using CNNs, aiming to handle the inadequate and inaccurate raw information mapping. Firstly, the bilinear feature was developed for tactile shapes to improve the feature extraction capabilities of CNN. The bilinear calculation of features is similar to the quadratic expansion of kernels [28,29]. The bilinear feature contains second-order characteristics of the tactile image that are sensitive to edge and texture information. Here, we use a single-mode CNN to generate bilinear features that allow the potential of the network itself to be fully exploited. Secondly, the multi-layer feature fusion strategy is applied to promote the feature utilization efficiency of CNN without increasing the network depth. The fused multi-layer features incorporate the advantages of both local and global information. Since the essence of CNN is a series of extractors stacked from low to high, and the obtained hierarchical features are the encoding of different layers of information [30], high-layer features describe semantic information but with fewer details, while low-layer features describe more detailed information but suffer from semantic ambiguity [31,32]. In this work, we use a skip connection to fuse the high-layer and low-layer features. The dimensionality of low-layer features is not reduced in the skip connection, and thus more complete details remain.

Therefore, a novel bilinear feature and multi-layer fused convolutional neural network (BMF-CNN) is proposed for tactile shape recognition. Letter shapes were chosen for performance verification because their contour is more complex than household objects and their non-ideal effects are more obvious. Tactile data were collected through a high-density sensor array. By comparing with the baseline traditional CNN and other manual design methods, the superiority of BMF-CNN was verified. We achieved an average recognition accuracy of 98.64%. This paper focuses on improving the recognition of tactile recognition limited by non-ideal effects. These non-ideal effects of density limitation and edge blurring caused by elastic coupling are unavoidable, and thus our method is equally suitable for other tactile perception tasks. Furthermore, the proposed framework can also further assist readers who intend to use CNNs for the task of inadequate mapping of raw information.

## 2. The Proposed BMF-CNN

### 2.1. Introduction to CNNs

**Figure 1:**
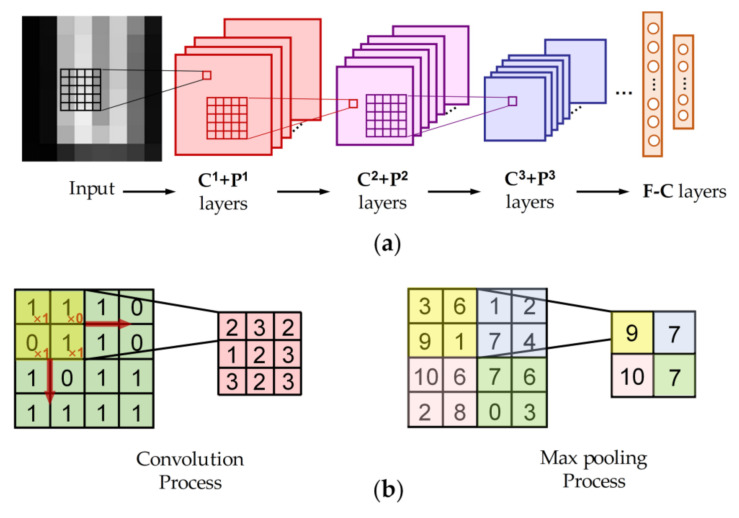
(**a**) The structure of a traditional convolutional neural network (CNN), (**b**) the process of convolution (kernel size: 2 × 2) and max pooling (pooling size: 2, stride: 2).

The CNNs consist of alternately configured convolutional layers and pooling layers for feature extraction and fully connected layers for forming semantic features. A traditional two-dimensional CNN and feature extraction process is shown in Figure 1. For convenience, we use **C** and **P** to represent the convolutional layer and pooling layer, respectively, and **F-C** for fully-connected layer. The convolution kernels are a set of filters, which generate feature maps by sweeping over the input. Mathematically, the *l*th output feature maps are calculated as:

(1) $$f^{l}=\psi (w^{l}⊗f^{l-1}+b^{l})$$

where *w* and *b* represent the convolutional kernels and bias, respectively, and *Ψ* (•) denotes the activation function, usually *ReLU* [33]. *ReLU* is described as:

(2) $$ReLU(x)=\left\{\begin{array}{c} x,\;if\;x>0\\ 0,\;if\;x\leq 0 \end{array}\right.$$

Because of the constant derivation (0 or 1), the gradient disappearance can be avoided and sparse activation is encouraged. The max pooling layer **P_max_** follows the **C** to perform the down-sampling operation to reduce the feature dimension, which is conducted by:

(3) maxpool(m,n)=max(α(s×m,s×n):α(s×m+k×m,s×n+k×n))

where *s* is the pooling stride. Concretely, the process is presented in Figure 1b. Maximum pooling preserves the maximum value within the window and therefore tends to preserve the highlighted textures. Finally, the distributed feature maps are represented to the label space by the **F-C**.

### 2.2. Single-Mode Bilinear Feature

**Figure 2:**
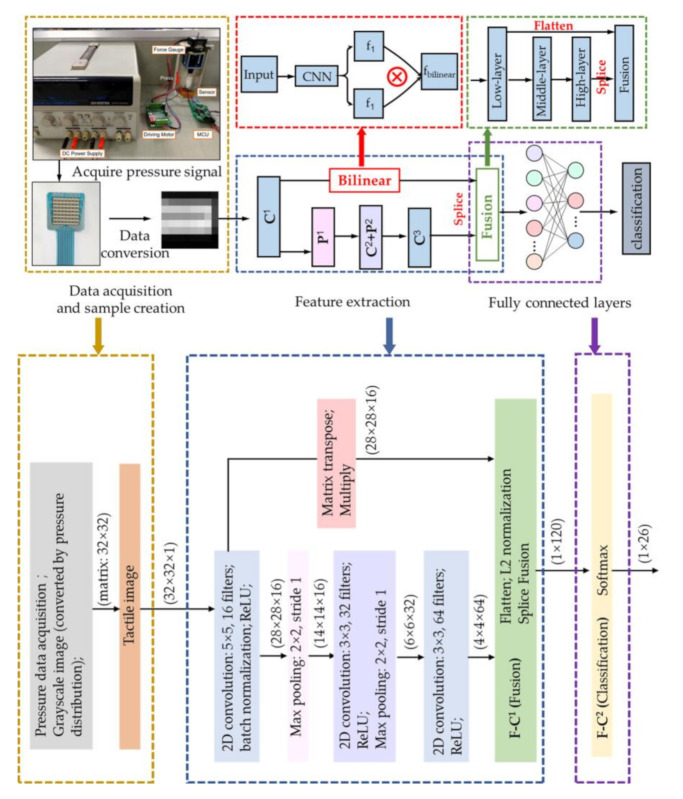
The framework of the proposed bilinear feature and multi-layer fused convolutional neural network (BMF-CNN).

The single-mode bilinear feature is constructed based on the matrix outer product strategy of the feature map extracted by the same convolutional neural network (red dotted box in Figure 2). The purpose is to represent the second-order characteristics of the tactile shape through expansion of the feature map, in a way similar to a quadratic kernel. These characteristics are very sensitive to edges and textures. Therefore, it is introduced in recognition of the low resolution of tactile shapes accompanied by elastic coupling, aiming to enhance the feature extraction capability of the network without increasing the depth.

The features extracted by CNN are represented as *f*, and the corresponding bilinear feature Φ is calculated as:

(4) $$\Phi =bilinear(f^{⊺}f)=\frac{1}{n}(\sum_{i=1}^{n}x_{1}x_{1}{}^{⊺})+\varepsilon I$$

This generates a covariance matrix that captures the pairwise interaction between the two features. Since the two features are the same, the obtained matrix is a symmetric positive semi-definite matrix. To further improve the bilinear feature representation, the above result was normalized using an elementwise signed square-root and followed by L2 norm regularization [29]:

(5) $$Z(f,f)=\frac{sign(\Phi )\times \sqrt{\Phi }}{{\left\|sign(\Phi )\times \sqrt{\Phi }\right\|}_{2}}$$

Due to the use of second-order features, the performance of single-mode bilinear CNN is better than the strategy of using first-order features for texture and edge-sensitive recognitions.

### 2.3. Multi-Layer Feature Fusion

Due to the blurring and low resolution of tactile images, the number and quality of the extracted features are limited. Hierarchical features are extracted by alternating convolution and pooling layers. Higher layer features contain mainly semantic information with less local detail, while lower layer features are more concerned with detailed information, but suffer from semantic ambiguity. How can the performance of the network be improved using limited features without increasing network depth? We can deal with this issue with an effective idea: the complementary strengths of features from different layers can be used. We suggest: (1) a high-utilization network should contain different information layers; (2) the combination of bilinear low-layer information and high-layer semantic information can better represent the characteristics of the tactile shape. Therefore, we developed a feature fusion strategy to feed multi-layer features into the network to improve feature utilization.

We apply skip connections to splice low layer features and high layer features for fusion (green dotted box in Figure 2). Feature maps of different layers have different sizes and channels, which cannot be directly fused. It is necessary to flatten the feature map into a 1D feature vector before fusion. If {h_1_ × h_1_/m} denotes the dimension of low-layer features and {h_2_ × h_2_/n} denotes the dimension of high-layer features, they are flattened into vectors of length h_1_ × h_1_ × m and h_2_ × h_2_ × n, respectively. The feature vectors are then spliced in the fusion layer as:

(6) $$f_{fusion}(f_{low},f_{high})=\psi ([f_{low},f_{high}])$$

whose lengths are h_1_ × h_1_ × m + h_2_ × h_2_ × n. Activation values of features from multi-layers vary widely, so the *L*_2_ normalization is used before the fusion to avoid features being overwritten. Since the splice operation does not require feature map dimensional matching, there is no dimension reduction of low-layer features. Hence, more complete local information is retained.

### 2.4. The BMF-CNN Framework

The proposed BMF-CNN framework is shown in Figure 2. First, the pressure data are converted into grayscale tactile images. Herein, the input size is 32 × 32 × 1. The BMF-CNN consists of three convolutional layers (**C^1^**, **C^2^**, **C^3^**), two max pooling layers (**P^1^_max_**, **P^2^_max_**) and two fully connected layers (**F-C^1^**, **F-C^2^**). The convolutional layers are used for feature extraction, and the kernels size here is {5 × 5/16}, {3 × 3/32}, and {3 × 3/64} with a stride of 1. Batch normalization [34] is added after **C^1^** to improve the convergence speed. The max pooling layer is applied when the convolution, batch normalization, and *ReLU* activation operations are complete, aiming to reduce the data dimensionality and further guarantee feature invariants, including translation and rotation. The pooling size here is 2 × 2 with a stride of 2. Two features are used for splice fusion: the low-layer features are bilinear computations of the features extracted by **C^1^** (bilinear **C^1^**), and the high-layer features are features extracted by **C^3^**. **F-C^1^** is used for feature splicing to obtain fused features (**f_fused_**) as an input to the classifier. A total of 120 neurons are set in **F-C^1^**, that is, the 1 × 1 convolutional kernel is applied to reduce the **f_fused_** to 1 × 120. The softmax classifier was chosen, which can directly classify multiple categories and select the category with the highest confidence score as:

(7) $$soft\max{\left(f\right)}_{i}=\frac{e^{f_{i}}}{\sum_{j}e^{f^{j}}}=P_{i}$$

This is consistent with the cross-entropy function in the backpropagation training, which is expressed by:

(8) $$Loss=-\sum_{i}f_{i}\log P_{i}$$

The details of the BMF-CNNs are shown in Table 1. The details of a baseline traditional CNN are also listed.

## 3. Experiment and Results

### 3.1. Dataset and BMF-CNN Training

**Figure 3:**
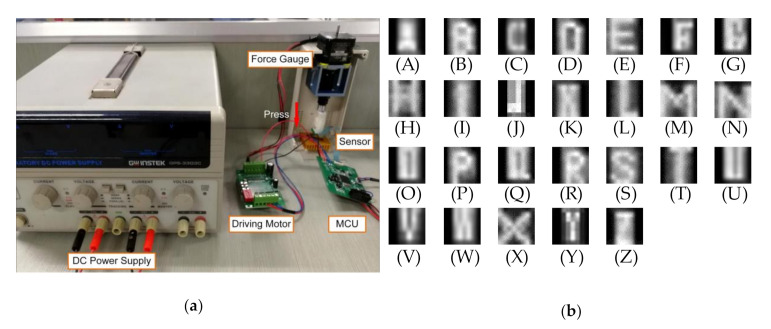
Self-made experimental system. (**a**) Pressure data acquisition system; (**b**) letter shape tactile image samples collected by this system.

As shown in Figure 3a, a self-made data acquisition system consisting of a sensor array, a force meter, a drive module, and a microcontroller unit was used for the experiment. A detailed description of the sensor array is shown in Table 2. The letter shapes were chosen because their shapes are more complex than those of household objects, which helps to verify the superiority of our proposed method. A stamp with letter-shaped apophysis was fixed to the force gauge and pressed on the sensor array. The output of the sensor array is a readout with a 20 kHz scanning frequency quantized and stored as a 32 × 32 matrix. The matrix is converted into a tactile grayscale image. A total of 500 samples per letter are collected within a random pressure range of 30–75 kPa, along with angle and position. To avoid overfitting, data augmentation is applied to expand the sample quantity to 1500 per letter category, including translation, random inversion, and contrast enhancement. These tactile images are divided into 26 categories marked A–Z according to the shape of the stamp. Figure 3b shows the data samples for each letter shape. Five-fold cross validation is used to divide the dataset for training and validation in the learning process. The BMF-CNN weights are initialized by the Xaiver initialization scheme and optimized by the Adam algorithm with hyperparameter parameters 0.9, 0.999, and ε = 10^‒8^ [35,36]. The learning rate was set to 0.001, batch size to 50, and the training epoch to 200. The experiment was implemented in the MatConvNet framework on a PC with an i5-5820 CPU@2.8 GHz.

### 3.2. Performance of the BMF-CNN

**Figure 4:**
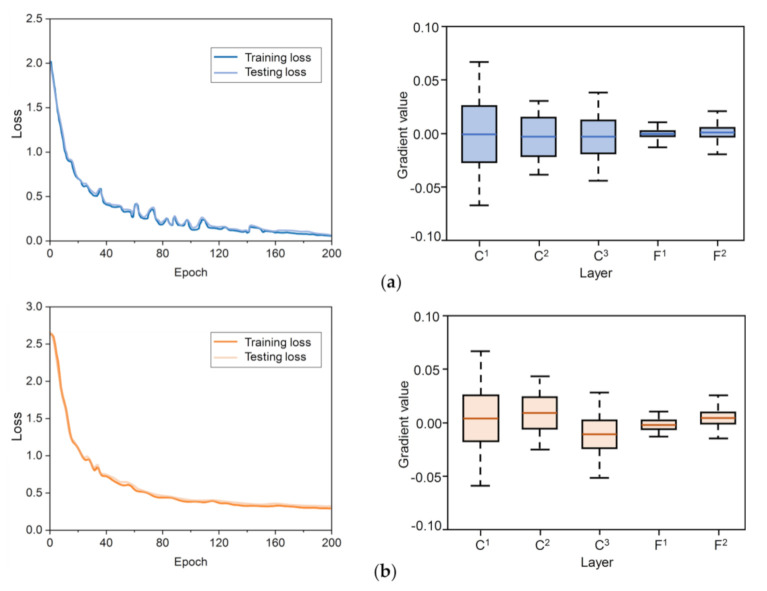
The training processes of the BMF-CNN and baseline CNN (traditional). (**a**) The losses of BMF-CNN and box plot of the gradients of the BMF-CNN; (**b**) the losses of traditional CNN and box plot of the gradients of the traditional CNN.

In the training processes, the losses and gradients of the BMF-CNNs are recorded in Figure 4a. The baseline traditional CNN (details in Table 1) was applied to the same dataset and the results are shown in Figure 4b. The losses of the network dropped rapidly before the 60th epoch, and no overfitting occurred. The gradient vanishing problem did not exist in the training processes of the BMF-CNN and traditional CNN, indicating that our dataset and model are reasonable and matched. It can be observed that the training losses and test losses of the BMF-CNN were lower than those of a traditional CNN. This demonstrates that our model is easy to train. 

**Figure 5:**
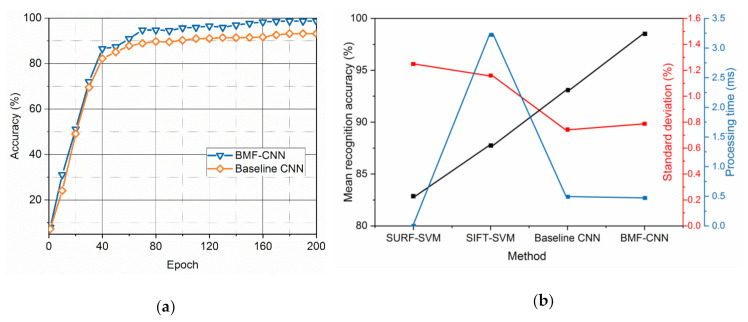
Recognition accuracy. (**a**) The test accuracy of the BMF-CNN and the baseline CNN on the same dataset; (**b**) performance comparison of SURF-SVM, SIFT-SVM, traditional CNN and BMF-CNN (mean recognition accuracy, standard deviation, and processing time).

Figure 5a shows the test accuracies using the BMF-CNN and the baseline CNN during 200 epochs. It can be observed that the BMF-CNN obtained the highest recognition accuracy of 98.66%, which is 5.45% higher than the 93.21% recognition accuracy of the traditional CNN. In addition to the traditional CNN, the SURF-SVM, and SIFT-SVM described in [7,8] were applied as a comparison benchmark to reveal the enhancement of the bilinearity and fusion strategies to the conventional CNN and manual design methods. The recognition accuracy of each method is illustrated in Figure 5b. In order to avoid randomness from affecting the experiment results, five trails were conducted for each method. The BMF-CNN achieved the highest accuracy of 98.64% with a 0.78% standard deviation, the convolutional CNN achieved 93.18% with a 0.73% standard deviation, and the two manual design methods of SURF-SVM and SIFT-SVM achieved 82.93% with a 1.31% standard deviation and 87.71% with a 1.23% standard deviation, respectively. The proposed method thus demonstrated excellent recognition performance and good stability. The recognition time of an image was computed to reveal the complexity of the network. Here we used the test processing time of a single tactile image as an indicator, which only includes the time of the previous propagation and classification of the network, excluding the processing time of back propagation and network parameter update. As shown in Figure 5b, SURF-SVM achieved the fastest recognition speed of 0.037 ms at the expense of recognition performance, while SIFT-SVM was the slowest at 3.2 ms, and BMF-CNN and the traditional CNN were 0.47 and 0.44 ms, respectively. The fast recognition speed shows that the performance of our BMF-CNN improved without entailing additional computational overhead, which is very important for practical applications. These results verify the effectiveness of the BMF-CNN to improve feature extraction ability and utilization efficiency. 

**Figure 6:**
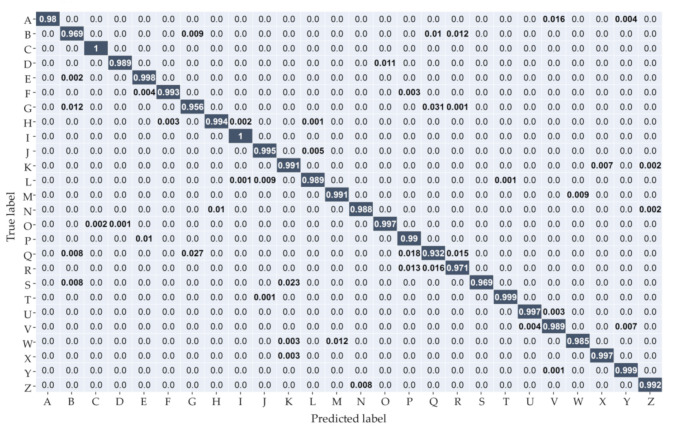
Confusion matrices of BMF-CNN.

To unravel the classification results of each tactile shape in detail, a confusion matrix with 26 classifications was drawn, as shown in Figure 6. It can be observed that letter shapes with simple outlines, such as C, I, O, and T, were easier to recognize and obtained higher accuracy. In particular, C and I both reached 100% accuracy. By contrast, samples with complex contours, such as G, R, Q, and B, were more likely to be misclassified due to low pixels and blurred edges. Some Q-shaped samples were erroneously classified as P, R, G, or D. Thus, the accuracy was only 93.2%. This can be attributed to the complex edges and spurious output caused by elastic coupling. Because the density of the sensor array is limited, the tactile image is low-resolution, which leads to insufficient and inaccurate mapping of the original information.

### 3.3. The Contribution of the Bilinear Features and Multi-Layer Fusion Strategies

**Figure 7:**
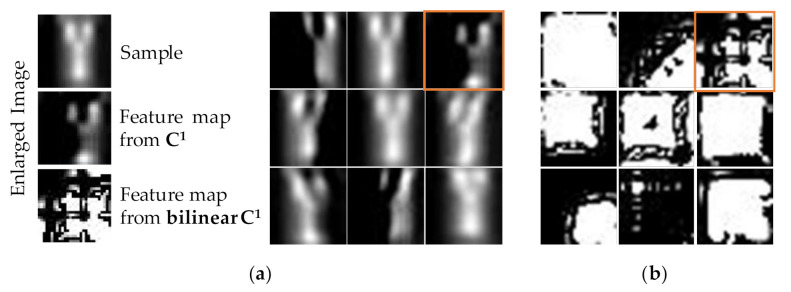
Feature map visualization. (**a**)the top-9 activating feature maps in **C^1^**. (**b**) the corresponding bilinear feature maps of **C^1^**. The enlarged images are marked by orange box.

In order to illustrate the contribution of bilinear features and fusion strategies to network performance in detail, we compared the recognition accuracy of different layers. For easy comparison, Table 3 reports the single-layer extracted feature accuracy. Obviously, the bilinear calculation significantly improved the separability of features. The accuracy increased by 16.5% from 45.6%. This result verifies that the bilinear features promote the feature extraction ability of the CNN. This is attributed to the fact that the bilinear calculation is similar to the feature expansion in the quadratic kernel. Due to the utilization of second-order characteristics, the single-mode BMF-CNN performed better on texture and edge-sensitive recognition tasks. To further reveal the bilinear features in detail, we chose to visualize the nine feature maps with max activation values obtained in **C^1^** after activating *ReLu* and corresponding bilinear feature maps. For a random sample (Y), Figure 7a shows the top nine feature maps in **C^1^** and Figure 7b shows the bilinear feature maps. It can be clearly observed that the bilinear features had significant symmetry. In addition, the difference between the feature maps was more obvious, which is of great significance to improve the separability of fusion features.

Our method aims to utilize the complementarity of high semantics and low local details. We explored the combination of different layers to reveal the effectiveness of the fusion strategy. The fusion operation was unified as follows: firstly, features from different layers are normalized by L2, then flattened, and finally spliced in **F-C^1^**. Here we tested the accuracy of **F-C^1^**. We used the accuracy of **F-C^1^** without fusion, obtained only by **C^3^**, as a benchmark for comparison. In addition, we also calculated the bilinear features extracted from **C^2^**. The results are reported in Table 4. Compared with no fusion, the multi-layer fusion strategy achieved better performance. The accuracy was promoted significantly, with a maximum increase of 4.5%. The improvement of the combination of direct fusion was less than the improvement of the combination of bilinear feature fusion. Moreover, the fusion combinations containing **C^1^** outperformed the corresponding fusion combinations containing **C^2^**. This is ascribed to the lower layer features describing more sufficient local information. Therefore, the fused features were more distinguishable.

**Figure 8:**
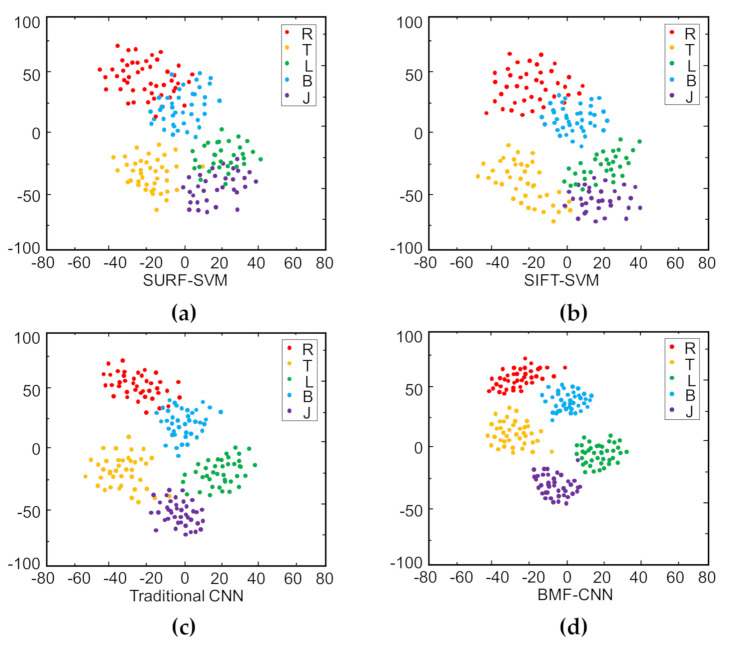
2D feature visualization using t-distributed random neighbor embedding (t-SNE): (**a**) SURF-SVM, (**b**) SIFT-SVM, (**c**) traditional CNN, (**d**) BMF-CNN.

To explain the superiority of bilinear features and multi-layer fusion more qualitatively, t-distributed random neighbor embedding (t-SNE) is used to visualize the learned features [37]. We used t-SNE to reduce the learned features in **F-C^1^** to two-dimensions. The mapped features for four methods are shown in Figure 8 (Five categories are randomly displayed, each containing 40 samples). In Figure 8, the shape features of different categories based on two artificially designs overlapped and did not cluster well, which matches the lower mean recognition accuracies shown in Figure 5b. For the traditional CNN, features of the same shape category were clustered, but slight overlap still occurred between different categories. By contrast, for BMF-CNN, the features within each category were tightly clustered and the features of different categories were widely spaced. This means that the features extracted by BMF-CNN have better separability, which is more intuitive for explaining the superiority of our proposed method.

## 4. Discussion

In this paper, a BMF-CNN using bilinear features and multi-layer fusion was proposed for low resolution and edge blurring tactile shape recognition. The bilinear feature is able to improve the ability of feature extraction so that the BMF-CNN can learn second-order characteristics better. Multi-layer fusion promotes feature utilization efficiency. For instance, the recognition accuracy is increased without deepening the network, as verified in Table 3 and Table 4. Due to non-ideal raw data, environmental interference, and other factors, very small and low-resolution images are common in practical recognition tasks. How to select the architecture of a CNN is still an open issue, and these problems are similar to the non-ideal effects of haptic images. Thus, we attempted to validate our framework on an additional dataset: *MNIST* [38], and achieved 99.21% accuracy (details are in the Supplementary Material). Our framework may enlighten us to handle other low-resolution tasks and we will focus on this topic in future work.

## 5. Conclusions

A framework called BMF-CNN was proposed for low-resolution and blurred edges tactile shape recognition. In this framework, the bilinear feature is developed to improve the neuron network feature extraction capability and the feature fusion strategy is applied to promoted the feature utilization capability to deal with low resolution and blurred edges. A 26 classes letter shape with complex edges dataset was used to verify the proposed BMF-CNNs. The recognition accuracy was 98.64%, which is much higher than that of traditional CNN and artificial design methods. Furthermore, we analyzed the contribution of bilinear features and fusion strategy to performance in detail through layer-by-layer accuracy verification and feature map visualization. 

## Figures and Tables

**Table 1:** Detailed parameters of BMF-CNN and baseline CNN (traditional).

Layer	BMF-CNN	CNN (Traditional)
Input	32 × 32 × 1	32 × 32 × 1
**C^1^**	5 × 5/16	5 × 5/16
Batch normalization (BN)	BN	BN
Bilinear	**28 × 28/16**	/
**P^1^**	2 × 2, stride 2	2 × 2, stride 2
**C^2^**	3 × 3/32	3 × 3/32
**P^2^**	2 × 2, stride 2	2 × 2, stride 2
**C^3^**	3 × 3/64	3 × 3/64
**F-C^1^**	120 **(Fusion)**	120 **(No Fusion)**
**F-C^2^**	26	26

**Table 2:** Parameter details of the sensor array applied in the experiment.

Parameter	Value
Pressure range	0–100 kPa
Temperature range	−25 to + 60 °C
Density	64 pixels/cm^2^
Sensitive pixel size	0.7 × 0.7 mm
Sensitive pixel repeatability	−2% to +6%
Number of signal lines	64
Sensor array height	5 mm
Sensor array height	5 mm
Sensor array thickness	0.1 mm

**Table 3:** The accuracy of different single layers.

Method	C^1^	Bilinear (C^1^)	C^2^	C^3^	F-C^1^
CNN (traditional)	0.457	/	0.646	0.807	0.908
BMF-CNN	0.456	0.637	0.647	0.807	0.953

**Table 4:** The accuracy of different combinations of two layers.

Layers	C^3^	Bilinear (C^1^) + C^3^	C^1^ + C^3^	Bilinear (C^2^) + C^3^	C^2^ + C^3^
**Accuracy**	0.908	**0.953**	0.927	0.931	0.916

## Supplementary Materials

The following are available online at https://www.mdpi.com/1424-8220/20/20/5822/s1, Figure S1: Examples of MNIST, Figure S2: Accuracy of BMF-CNN on MNIST, Table S1: Performance comparison on MNIST.

## References

[1] Choi S., Lee H., Ghaffari R., Hyeon T., Kim D.H. (2016). Recent Advances in Flexible and Stretchable Bio-Electronic Devices Integrated with Nanomaterials. Adv. Mater..

[2] Yang J.C., Mun J., Kwon S.Y., Park S., Bao Z.N., Park S. (2019). Electronic Skin: Recent Progress and Future Prospects for Skin-Attachable Devices for Health Monitoring, Robotics, and Prosthetics. Adv. Mater..

[3] Chortos A., Liu J., Bao Z.A. (2016). Pursuing prosthetic electronic skin. Nat. Mater..

[4] Pezzementi Z., Plaku E., Reyda C., Hager G.D. (2011). Tactile-Object Recognition from Appearance Information. IEEE Trans. Robot.

[5] Suto S., Watanabe T., Shibusawa S., Kamada M. (2018). Multi-Touch Tabletop System Using Infrared Image Recognition for User Position Identification. Sensors.

[6] Gastaldo P., Pinna L., Seminara L., Valle M., Zunino R. (2014). A Tensor-Based Pattern-Recognition Framework for the Interpretation of Touch Modality in Artificial Skin Systems. IEEE Sens. J..

[7] Luo S., Mou W.X., Althoefer K., Liu H.B. (2015). Novel Tactile-SIFT Descriptor for Object Shape Recognition. IEEE Sens. J..

[8] Gandarias J.M., Gomez-de-Gabriel J.M., Garcia-Cerezo A. Human and Object Recognition with a High-Resolution Tactile Sensor. Proceedings of the 2017 IEEE Sensor.

[9] Khasnobish A., Jati A., Singh G., Konar A., Tibarewala D.N. (2014). Object-Shape Recognition by Tactile Image Analysis Using Support Vector Machine. Int. J. Pattern. Recogn..

[10] Luo S., Bimbo J., Dahiya R., Liu H.B. (2017). Robotic tactile perception of object properties: A review. Mechatronics.

[11] Li P.X., Wang D., Wang L.J., Lu H.C. (2018). Deep visual tracking: Review and experimental comparison. Pattern Recogn..

[12] Voulodimos A., Doulamis N., Doulamis A., Protopapadakis E. (2018). Deep Learning for Computer Vision: A Brief Review. Comput. Intel. Neurosc..

[13] Qi C.R., Su H., Mo K.C., Guibas L.J. PointNet: Deep Learning on Point Sets for 3D Classification and Segmentation. Proceedings of the 2017 IEEE Conference on Computer Vision and Pattern Recognition (CVPR).

[14] Zhu Z.T., Wang X.G., Bai S., Yao C., Bai X. (2016). Deep Learning Representation using Autoencoder for 3D Shape Retrieval. Neurocomputing.

[15] Gandarias J.M., Garcia-Cerezo A.J., Gomez-de-Gabriel J.M. (2019). CNN-Based Methods for Object Recognition With High-Resolution Tactile Sensors. IEEE Sens. J..

[16] Pastor F., Gandarias J.M., Garcia-Cerezo A.J., Gomez-de-Gabriel J.M. (2019). Using 3D Convolutional Neural Networks for Tactile Object Recognition with Robotic Palpation. Sensors.

[17] Cao L.L., Sun F.C., Liu X.L., Huang W.B., Kotagiri R., Li H.B. (2018). End-to-End ConvNet for Tactile Recognition Using Residual Orthogonal Tiling and Pyramid Convolution Ensemble. Cogn. Comput..

[18] Tsuji S., Kohama T. (2019). Using a convolutional neural network to construct a pen-type tactilesensor system for roughness recognition. Sens. Actuators A Phys..

[19] Hui W., Li H., Chen M., Song A. (2019). Robotic tactile recognition and adaptive grasping control based on CNN-LSTM. Chin. J. Entific. Instrum..

[20] Funabashi S., Yan G., Geier A., Schmitz A., Ogata T., Sugano S. Morphology-Specific Convolutional Neural Networks for Tactile Object Recognition with a Multi-Fingered Hand. Proceedings of the 2019 IEEE Conference on Robotics and Automation (ICRA).

[21] Alameh M., Valle M., Ibrahim A., Moser G. DCNN for Tactile Sensory Data Classification based on Transfer Learning. Proceedings of the 2019 15th Conference on Ph.D Research in Microelectronics and Electronics (PRIME).

[22] Wang S.H., Xu J., Wang W.C., Wang G.J.N., Rastak R., Molina-Lopez F., Chung J.W., Niu S.M., Feig V.R., Lopez J. (2018). Skin electronics from scalable fabrication of an intrinsically stretchable transistor array. Nature.

[23] He K.M., Zhang X.Y., Ren S.Q., Sun J. Deep Residual Learning for Image Recognition. Proceedings of the 2016 IEEE Conference on Computer Vision and Pattern Recognition (CVPR).

[24] Simonyan K., Zisserman A. (2014). Very Deep Convolutional Networks for Large-Scale Image Recognition. arXiv.

[25] Brahimi S., Ben Aoun N., Ben Amar C. Improved Very Deep Recurrent Convolutional Neural Network for Object Recognition. Proceedings of the 2018 IEEE International Conference on Systems, Man, and Cybernetics (SMC).

[26] Chu J., Cai J.P. (2020). Flexible pressure sensors with a highly pressure- and strain-sensitive layer based on nitroxyl radical-grafted hollow carbon spheres. Nanoscale.

[27] Li F.Y., Akiyama Y., Wan X.L., Okamoto S., Yamada Y. (2020). Measurement of Shear Strain Field in a Soft Material Using a Sensor System Consisting of Distributed Piezoelectric Polymer Film. Sensors.

[28] Lin T.Y., RoyChowdhury A., Maji S. Bilinear CNN Models for Fine-grained Visual Recognition. Proceedings of the 2015 IEEE International Conference on Computer Vision (ICCV).

[29] Zhang W.X., Ma K.D., Yan J., Deng D.X., Wang Z. (2020). Blind Image Quality Assessment Using a Deep Bilinear Convolutional Neural Network. IEEE Trans. Circ. Syst. Vid..

[30] Hariharan B., Arbeláez P., Girshick R., Malik J. Hypercolumns for Object Segmentation and Fine-grained Localization. Proceedings of the 2015 IEEE Conference on Computer Vision and Pattern Recognition (CVPR).

[31] Yu W., Yang K.Y., Yao H.X., Sun X.S., Xu P.F. (2017). Exploiting the complementary strengths of multi-layer CNN features for image retrieval. Neurocomputing.

[32] Sermanet P., LeCun Y. Traffic Sign Recognition with Multi-Scale Convolutional Networks. Proceedings of the 2011 International Joint Conference on Neural Networks (IJCNN).

[33] Glorot X., Bordes A., Bengio Y. Deep Sparse Rectifier Neural Networks. Proceedings of the 14th International Conference on Artificial Intelligence and Statistics (AISTATS).

[34] Ioffe S., Szegedy C. Batch Normalization: Accelerating Deep Network Training by Reducing Internal Covariate Shift. Proceedings of the International Conference on Machine Learning (ICML).

[35] Kingma D., Ba J. Adam: A method for stochastic optimization. Proceedings of the International Conference on Learning Representations (ICLR).

[36] Glorot X., Bordes A. Understanding the difficulty of training deep feedforward neural networks. Proceedings of the 13th International Conference on Artificial Intelligence and Statistics (AISTATS), Chia Laguna Resort.

[37] Laurens V.D.M., Hinton G. (2008). Visualizing data using t-SNE. J. Mach. Learn. Res..

[38] Lecun Y. MNIST Handwritten Digit Database. http://yann.lecun.com/exdb/mnist/.

