# Supplementary material for: A Novel Bilinear Feature and Multi-Layer Fused Convolutional Neural Network for Tactile Shape Recognition
Source: Sensors (Basel). 2020 Oct 15;20(20):5822. doi: 10.3390/s20205822 (PMC7602476; doi:10.3390/s20205822)
Supplement: Supplementary file 1 [file sensors-20-05822-s001.pdf]

Supporting Material

Validation of BMF-CNN on the MNIST Dataset

Step 1: Dataset Pre-Processing

To validate the generalization performance of the BMF-CNN, the MNIST [1] dataset was used as a validation benchmark. The MNIST dataset is a fundamental dataset in the field of target recognition and image classification, which consists of handwritten numbers from 0 to 9. The dataset has a total of 70,000 samples, of which 60,000 (approximately 6000 per number) are training samples and 10,000 are test samples. Since the samples in MNIST are stored in a size of 28 × 28, in order to meet the requirements of the input image size of the BMF-CNN, the MNIST image needs to be extended. Here we used 0 padding, and the specific mathematical operation is as follows:

$$O(32,32) = (\{0,0,I,0,0\},\{0,0,I,0,0\})$$

where O is the expanded matrix and I denotes the original matrix.

Step 2: Training Process

Algorithm 2

Input:
dataset and corresponding label D={x_m,y_m | m=1,2…M}; batch size=50;
Process:
  Xavier initial;
  repeat:
    for each{x_m,y_m | m=1,2…batchsize} **in** D **do**
      $y_{logists}$ ←**softmax**(full connection(y_k))
      calculate the cross-entropy loss function with $y_{logists}$
      loss back propagation
      update network weight with **Adam**
    end for
  until training epoch;
return complete training.

Step 3: Performance Analysis of BMF-CNN

The proposed BMF-CNN framework reached an accuracy of 99.21%, and the training processeses are shown in Figure S2. Additionally, we compared some state-of-art and classical methods on the MNIST, and the recognition performance is shown in Table S1. It can be observed that our framework reached the highest accuracy, which is higher than that of the tactile image dataset. The reasons may be as following: (1) The MNIST dataset is more complete, with 6000 images in each category, so it has sufficient data to learn; (2) the images in the MNIST are 2-valued (Figure S1), with only 0 or 255, whereas the tactile image is grayscale, with the range of [0,255]. The features of the image in MNIST are more obvious.

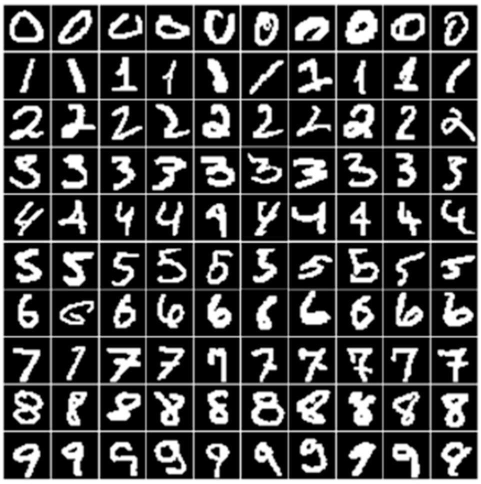

Figure S1. Examples of MNIST.

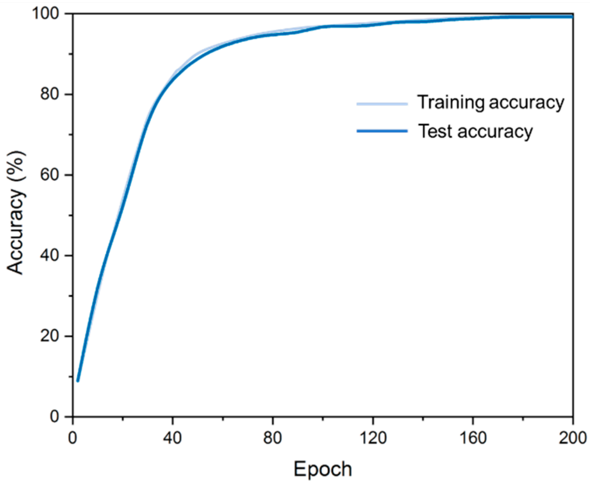

Figure S2. Accuracy of BMF-CNN on MNIST.

Table S1. Performance comparison on MNIST.

Method	Reference	Accuracy	Processing Time (ms)
BMF-CNN	This paper	99.21%	0.45
Traditional CNN	This paper	98.83%	0.41
Lenet-5	[2]	99.05%	0.39
CNN-SVM	[3]	99.10%	0.44
SURF-SVM	[4]	96.75%	0.033

Reference:

1. Lecun, Y. MNIST Handwritten Digit Database. Available Online: http://yann.lecun.com /exdb/mnist/ (accessed 22 September 2020)
2. Lecun, Y.; Bottou, L.; Bengio, Y.; Haffner P. Gradient-based learning applied to document recognition. *Proc. IEEE* **1998**, *86*, 2278–2324.
3. Maitra, D.; Bhattacharya, S.; Parui, S.K. CNN based common approach to handwritten character recognition of multiple scripts. In Proceedings of the 13th International Conference on Document Analysis and Recognition (ICDAR), Tunis, Tunisia, 23–26 August 2015; pp. 1021–1025.
4. Gandarias, J.M.; Gomez-de-Gabriel, J.M.; Garcia-Cerezo, A. Human and Object Recognition with a High-Resolution Tactile Sensor. In Proceedings of the IEEE Sensor, Glasgow, UK, 29 October–1 November 2017; pp. 981–983.
